# Ascending complex anal fistula secondary to lower extremity soft tissue infection: a case report

**DOI:** 10.3389/fsurg.2026.1823865

**Published:** 2026-04-30

**Authors:** Jiyuan Zhang, Xiaopeng Ma, Liangchen Li, Xiaoning Qin, Leiran Feng, Guiying Wang

**Affiliations:** Department of General Surgery, The Second Hospital of Hebei Medical University, Shijiazhuang, China

**Keywords:** case report, complex anal fistula, fistulography, MRI, perianal abscess

## Abstract

**Background:**

Complex anal fistulas remain a surgical challenge due to their intricate anatomy, high recurrence rates, and potential for sphincter injury. We report a rare case of complex anal fistula with multiple external openings extending from the gluteal region to the thigh root, successfully managed by combined incision with drainage tube placement.

**Case presentation:**

A 57-year-old man presented with recurrent purulent discharge from the perianal region and left lower limb for three months following debridement of a lower extremity soft tissue infection. Physical examination revealed multiple unhealed wounds on the left buttock, thigh root, and lower limb with purulent exudation. A cord-like sinus tract was palpable in the left perianal region extending into the anal canal and communicating with the buttock wound. Pelvic contrast-enhanced MRI demonstrated multiple enhancing sinus tracts within the left gluteal subcutaneous tissue and gluteus maximus muscle, extending superomedially to the posterior anal aspect and inferolaterally along the muscle surface to the dorsal thigh root. Fistulography confirmed communication between the left buttock fistula and the rectum, with contrast entering the rectal lumen approximately 2.0 cm from the anal verge. Colonoscopy revealed two NICE type 2 polyps in the descending and sigmoid colon (3 mm × 3 mm and 6 mm × 7 mm), which were removed by endoscopic mucosal resection; pathology confirmed tubular adenomas. The patient underwent fistulotomy with drainage tube placement for the high trans-sphincteric tract extending to the supralevator space, along with partial sinus tract excision and drainage tube insertion. Intraoperative blood loss was approximately 10 mL. The postoperative course was uneventful, and at six-month follow-up, the wounds had completely healed with no signs of recurrence and normal anal sphincter function.

**Conclusion:**

This rare case of complex anal fistula with multiple external openings and deep extension to the thigh root highlights the critical role of multimodal imaging (MRI and fistulography) in preoperative planning. Combined incision, drainage, and drainage tube placement achieved successful outcomes with preserved sphincter function.

## 1 Introduction

Anal fistula is an abnormal epithelialized tract connecting the anorectum to the perianal skin. In most cases, the source is considered to be a non-specific cryptoglandular infection starting from the intersphincteric space (1). Complex anal fistulas are generally defined by their involvement above the levator ani muscle, the presence of multiple external openings, secondary extensions or branching, or association with underlying conditions such as Crohn's disease (2). These fistulas present significant therapeutic challenges due to their high recurrence rates and the risk of postoperative incontinence. Preoperative imaging, particularly magnetic resonance imaging (MRI) and fistulography, plays an indispensable role in accurately delineating fistula anatomy and guiding surgical strategy. We present a rare case of complex anal fistula resulting from soft tissue infection of the lower extremity. This fistula features multiple external openings and extends from the gluteal region to the root of the thigh. Additionally, we discuss the diagnosis and surgical management of this case.

## 2 Case presentation

A 57-year-old man was admitted to our department with a three-month history of recurrent purulent discharge from the perianal region and left lower limb. The patient first visited the hospital due to swelling and pain of the left lower limb with fever 3 months ago. MRI showed multiple muscle swelling in the left lower limb, accompanied by multiple gas and fluid accumulation in the subcutaneous and intermuscular space, which was diagnosed as soft tissue infection of the left lower limb (Supplementary Material 1). Subsequently, the patient underwent debridement of the left thigh and lower leg and flap grafting in the burn department (Supplementary Material 2). However, perianal discharge was first noted shortly after the debridement procedure, surgery for perianal symptoms was not performed immediately. After waiting for three months, the patient's lower limb wound initially healed, and then visited our department for treatment of anal symptoms. During this period, the patient did not receive any relevant treatment. His medical history included a traumatic amputation of the distal phalanges of the right index. He denied any history of hypertension, diabetes, coronary heart disease, hepatitis, tuberculosis, or inflammatory bowel disease. Family history: no history of anal fistula, IBD, or colorectal cancer. Psychosocial history: patient is a manual worker, non-smoker, non-drinker. Genetic information: no suspicion of hereditary polyposis syndromes.

The patient initially presented with left lower limb soft tissue infection three months prior to admission, for which he underwent debridement. The perianal purulent discharge did not precede the limb infection; rather, it developed approximately after debridement and persisted despite wound care. This temporal sequence raises the possibility of ascending spread of infection from the thigh to the perianal and perirectal regions, rather than the classic cryptoglandular pathway.

Physical examination in the knee-chest position revealed multiple unhealed wounds on the left buttock, left thigh root, and left lower limb, with purulent exudation upon compression. A palpable cord-like sinus tract was identified in the left perianal region, extending into the anal canal and communicating with the buttock wound. Digital rectal examination revealed tenderness and a vague sense of fluctuation, but no distinct mass was palpable.

Colonoscopy identified two polyps in the descending colon (3 mm × 3 mm) and sigmoid colon (6 mm × 7 mm), both classified as NICE type 2. Endoscopic mucosal resection (EMR) was performed, and the resection sites were closed with titanium clips. Pathological examination of the polyps confirmed tubular adenomas. The rectal mucosa showed scattered erythema and tortuous vessels, but no fistula opening was visualized. Colonoscopy effectively ruled out Crohn's disease and malignancy.

**Figure 1:**
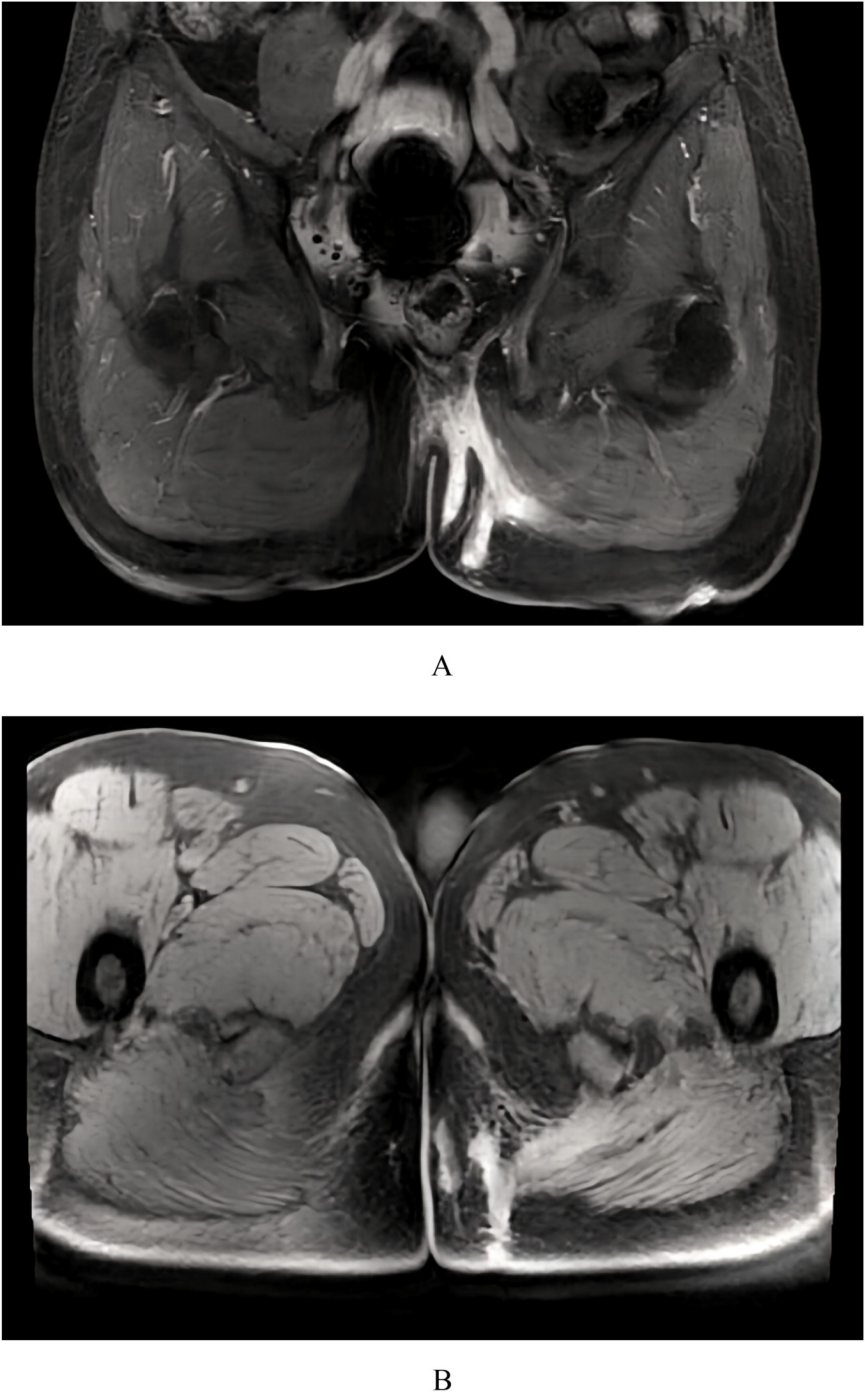
Pelvic contrast-enhanced MRI. Coronal **(A)** and axial **(B)** views demonstrating enhancing sinus tracts extending from the left gluteal region through the gluteus maximus muscle to the dorsal aspect of the left thigh root.

Pelvic contrast-enhanced MRI revealed multiple enhancing sinus tracts within the left gluteal subcutaneous tissue and the gluteus maximus muscle. These tracts extended superomedially to the posterior aspect of the anal canal and inferolaterally along the muscle surface to the dorsal aspect of the left thigh root, with the external opening located on the posterior thigh skin. Surrounding inflammatory changes were noted (Figure 1).

**Figure 2:**
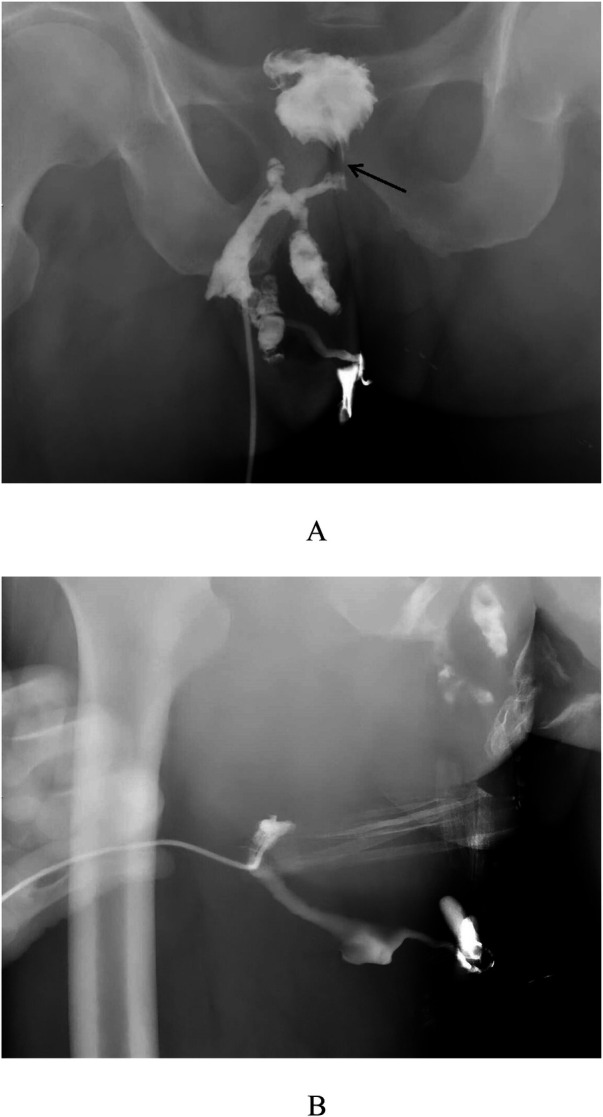
Fistulography. Injection of contrast through the left buttock external opening **(A)** demonstrates a branching tract communicating with the rectum (arrow). Injection through the left thigh root opening **(B)** shows localized contrast opacification without communication.

Fistulography was performed by injecting iohexol through the left buttock fistula opening. This demonstrated multiple branching tracts within the left buttock region, with one tract clearly communicating with the rectum; contrast agent was observed entering the rectal lumen approximately 2.0 cm from the anal verge. Injection through the left thigh root fistula opening showed localized contrast opacification without communication with other tracts (Figure 2).

**Figure 3:**
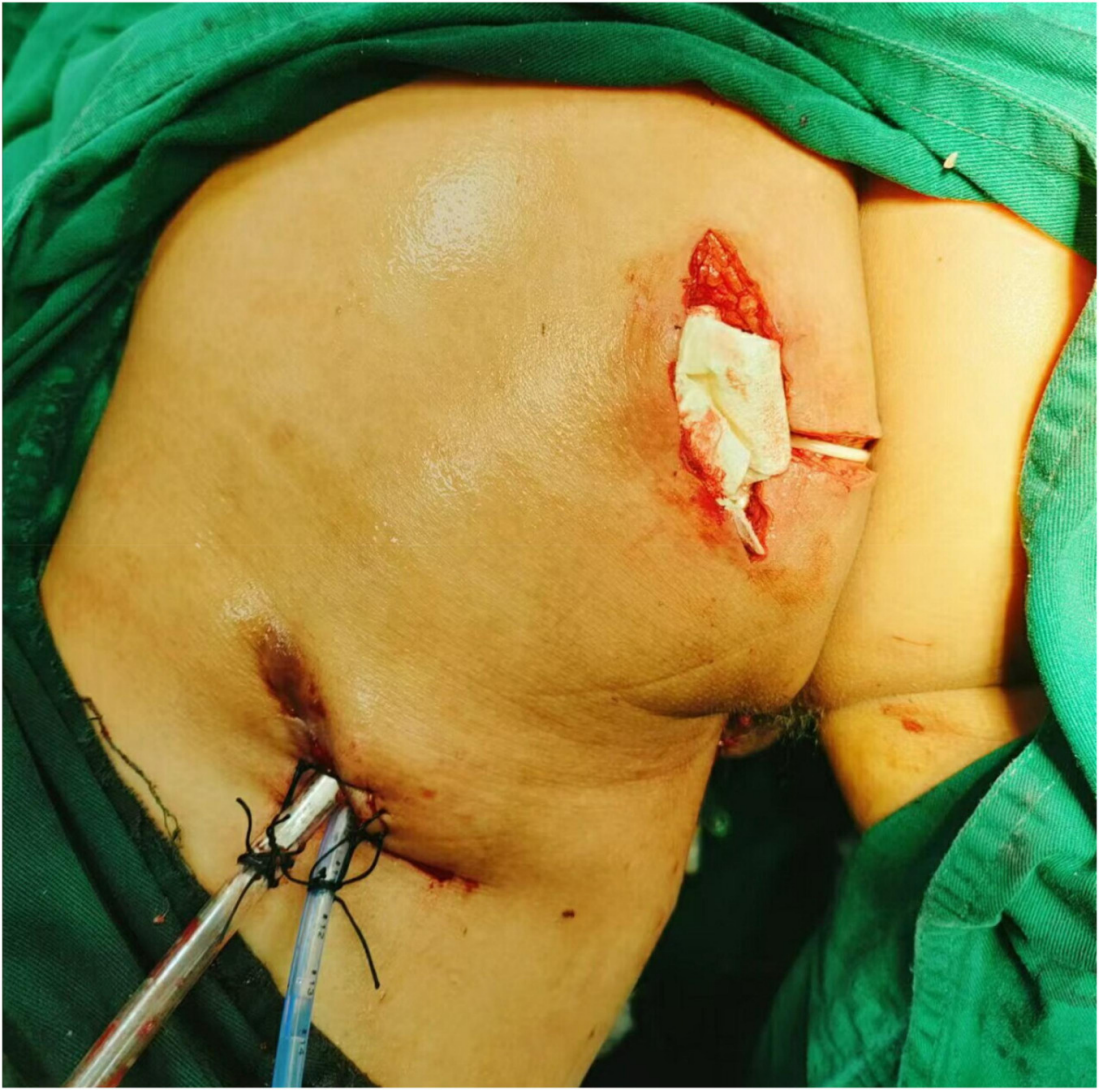
The postoperative incision and the placement location of the drainage tube.

The main challenge in treating this case lies in the excessively long fistula that has spread to the lower limbs. The most crucial issue is to minimize the exposure damage and address the patient's infection. The patient underwent surgery under general anesthesia in the prone jackknife position. Following routine disinfection and anal dilatation, a skin fold approximately 3 cm in diameter with purulent discharge was identified at the 9 o'clock position, about 3 cm from the anal verge. Injection of diluted methylene blue solution through this opening resulted in blue staining within the anal canal, confirming the internal opening. Probe exploration revealed that the sinus tract passed through the levator ani muscle into the supralevator space and communicated with the 0.3 cm diameter external opening at the thigh root. The external opening at the anal verge was incised along the probe direction, and a portion of the anorectal muscle was excised. The internal opening was identified at the 11 o'clock position near the dentate line. A partial sinus tract excision was performed, and a cutting seton (rubber band) was placed through the high trans-sphincteric. Based on the patient's condition, the first tightening of the drain was performed one week after the surgery. A drainage tube was inserted into the highest point of the sinus cavity. Debridement of the tract from the thigh root to the perianal region was performed, and a second drainage tube was placed and exteriorized through the thigh root opening. The surgical field was irrigated with hydrogen peroxide and normal saline, and meticulous hemostasis was achieved. Intraoperative blood loss was approximately 10 mL, and no blood transfusion was required. The excised fistula tissue was sent for histopathological examination (Figure 3).

Histopathology of the sinus tract revealed fibroadipose tissue with striated muscle, lined by stratified squamous epithelium. Subepithelial fibrous tissue proliferation, multifocal chronic inflammatory cell infiltration, granulation tissue formation, fresh and old hemorrhage, focal tissue degeneration and necrosis with acute inflammatory exudate, and multifocal multinucleated giant cell reaction were observed, consistent with a sinus tract.

**Figure 4:**
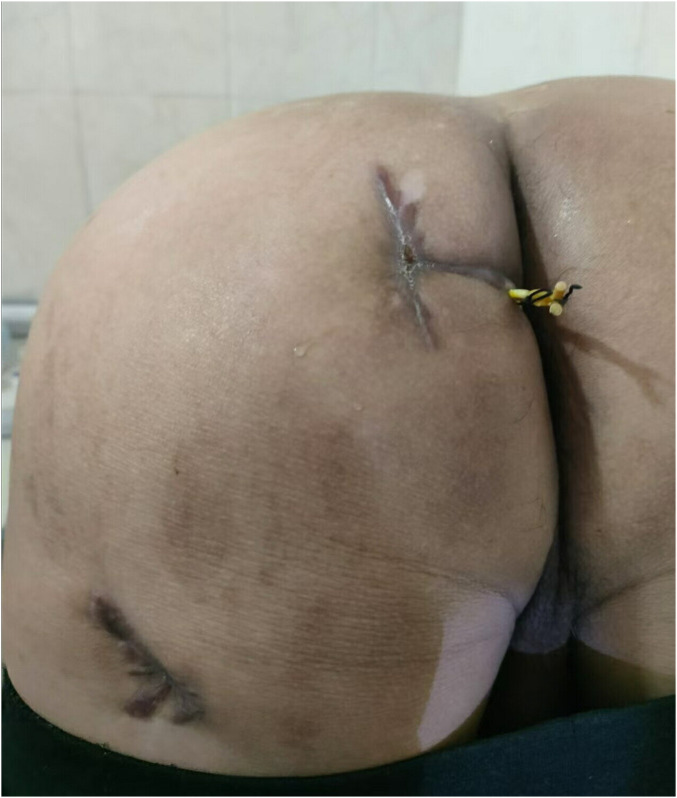
The patient's image taken two months after the surgery. The drainage tube has been removed and the incision has healed well.

**Figure 5:**
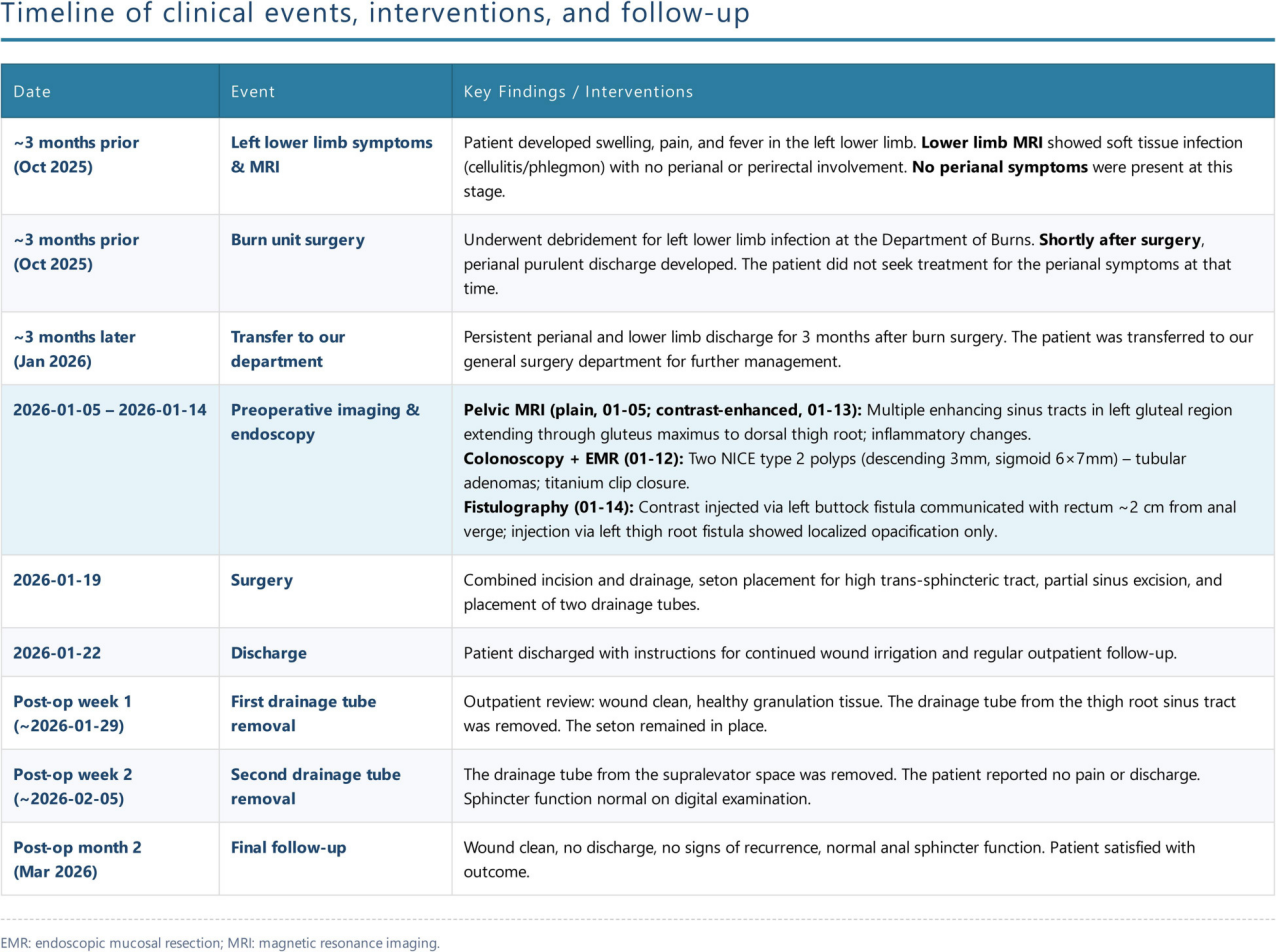
The timeline of clinical events and interventions.

The patient's postoperative recovery was uneventful, with no fever, abdominal pain, or distension, and normal bowel and bladder function. The surgery resolved the patient's symptoms of perianal discomfort and continuous pus discharge, removed the fistula that extended to the leg, and did not affect the patient's anal function. The patient was quite satisfied with the result and no adverse events occurred. He was discharged with instructions for continued wound irrigation. At 2-month follow-up (outpatient clinic and telephone contact), the patient received regular follow-up and medication changes. The drainage tube was gradually removed. The patients had two drainage tubes removed during two follow-up dressing changes in the first and second week after surgery (Figure 4). The timeline of the entire disease is shown in the figure (Figure 5).

## 3 Discussion

Based on our clinical experience, the initial symptoms of the patient were swelling and pain in the left lower limb along with fever. From the initial symptoms and the MRI results, there was no evidence of the original condition of anal fistula. Moreover, after the surgery, the patient developed perianal symptoms. Therefore, we suspect that the infection in the lower limb soft tissues spread to the perianal area.

This case presents a rare and complex anal fistula characterized by: (1) the infection in the lower limb soft tissues spread to the perianal area. (2) An unusually long tract extending from the buttock through the gluteus maximus muscle to the thigh root; (3) the presence of multiple external openings (buttock and thigh root); (4) associated colonic polyps without evidence of inflammatory bowel disease; and (5) a clear communication with the rectum, with the internal opening located near the dentate line.

MRI was instrumental in the preoperative evaluation, precisely delineating the relationship of the fistula tracts to the surrounding musculature and the ischioanal fossa, and excluding deep abscesses or unsuspected extensions (3). Fistulography provided complementary dynamic information by confirming the communication between the buttock fistula and the rectum, which directly guided the surgical approach.

The surgical strategy employed—a combination of incision, drainage, and drainage tube placement—represents a well-established approach for high trans-sphincteric fistulas. Surgical management of a fistula is focused on preservation of continence while achieving healing (4). Drainage tube placement allows for gradual, controlled division of the sphincter muscle while promoting fibrosis, thereby reducing the risk of incontinence associated with complete primary fistulotomy (5). In our patient, complete excision of the deep supralevator extension would have carried an unacceptable risk of sphincter damage; therefore, drainage tube placement combined with partial excision and adequate drainage was the optimal choice. The uneventful healing and preserved sphincter function at six months confirm the appropriateness of this approach.

The incidental finding of colonic tubular adenomas in this patient, while not etiologically related to the anal fistula, raises an important consideration. Patients with complex anal fistulas, particularly older individuals, may benefit from routine colonoscopic screening to exclude synchronous colorectal pathology (6). This is consistent with broader recommendations for comprehensive evaluation in patients with fistulous disease (7).

For such sinus tracts that spread from the lower limbs to the buttocks, we avoided performing extensive incisions for draining and instead chose to gradually remove the drainage tubes to facilitate the closure of the tracts. This individualized treatment avoided exposing larger wounds and alleviated the postoperative pain of the patient. Although like new technologies such as fibrin glues, dermal collagen injection, the anal fistula plugs, and stem cell injection offer alternative approaches, individualized treatment remains the most important option for treating anal fistulas (8).

Several features of this case merit emphasis. First, the extension of a fistula tract to the thigh root is distinctly uncommon and should be considered in the differential diagnosis of non-healing sinus tract in the lower extremity. Second, the combination of MRI and fistulography provided complementary information that was essential for accurate surgical planning (9). Third, this case demonstrates that a sphincter-preserving approach using drainage tube placement can achieve excellent outcomes even in very extensive fistulas.

In this case, we also have areas that need improvement. The patient did not come to our department for treatment immediately after the symptoms appeared, which led to a delay in the treatment of the condition. Timely referral can further optimize the treatment outcome (10). We lacked the earliest preoperative photos for reference and comparison. Considering the patient's financial situation after the surgery and the fact that the patient's main symptoms had disappeared, no imaging examination was conducted on the patient.

## 4 Conclusion

This case report describes a rare complex anal fistula with multiple external openings and deep extension to the thigh root, successfully managed with a combination of incision, drainage, and drainage tube placement. The case underscores the critical importance of multimodal imaging (MRI and fistulography) in accurately defining fistula anatomy and guiding individualized surgical planning. It also highlights the value of comprehensive gastrointestinal evaluation in patients presenting with complex fistulous disease.

## Data Availability

The original contributions presented in the study are included in the article/Supplementary Material, further inquiries can be directed to the corresponding author.

## References

[1] Amato A Bottini C De Nardi P Giamundo P Lauretta A Realis Luc A Evaluation and management of perianal abscess and anal fistula: SICCR position statement. Tech Coloproctol. (2020) 24(2):127–43. 10.1007/s10151-019-02144-1 31974827

[2] Yamamoto T Kotze PG Spinelli A Panaccione R. Fistula-associated anal carcinoma in Crohn’s disease. Expert Rev Gastroenterol Hepatol. (2018) 12(9):917–25. 10.1080/17474124.2018.1500175 29999429

[3] Bartram C Buchanan G. Imaging anal fistula. Radiol Clin North Am. (2003) 41(2):443–57. 10.1016/S0033-8389(02)00122-7 12659348

[4] Gandhi ND Alibo EO Gipe JH. Anal Abscess and Fistula. Surg Clin North Am. (2026) 106(1):51–63. 10.1016/j.suc.2025.08.012 41241453

[5] Bhat S Xu W Varghese C Dubey N Wells CI Harmston C Efficacy of different surgical treatments for management of anal fistula: a network meta-analysis. Tech Coloproctol. (2023) 27(10):827–45. 10.1007/s10151-023-02845-8 37460830 PMC10485107

[6] Mei Z Wang Q Zhang Y Liu P Ge M Du P Risk factors for recurrence after anal fistula surgery: a meta-analysis. Int J Surg. (2019) 69:153–64. 10.1016/j.ijsu.2019.08.003 31400504

[7] Feroz SH Ahmed A Muralidharan A Thirunavukarasu P. Comparison of the efficacy of the various treatment modalities in the management of perianal Crohn’s fistula: a review. Cureus. (2020) 12(12):e11882. 10.7759/cureus.11882 33415035 PMC7781784

[8] Cadeddu F Salis F Lisi G Ciangola I Milito G. Complex anal fistula remains a challenge for colorectal surgeon. Int J Colorectal Dis. (2015) 30(5):595–603. 10.1007/s00384-014-2104-7 25566951

[9] Zubaidi AM. Anal fistula. Past and present. Saudi Med J. (2014) 35(9):937–44. 25228174

[10] Zhang M Wang W Xia Y Zhang H Du Y Wang Z Management of complex anal fistula in recurrent perianal abscess: a case report. Am J Case Rep. (2025) 26:e948682. 10.12659/AJCR.948682 40946157 PMC12445932

