# Supplementary figures and images for: Ascending complex anal fistula secondary to lower extremity soft tissue infection: a case report
Source: Front Surg. 2026 Apr 30;13:1823865. doi: 10.3389/fsurg.2026.1823865 (PMC13171473; doi:10.3389/fsurg.2026.1823865)

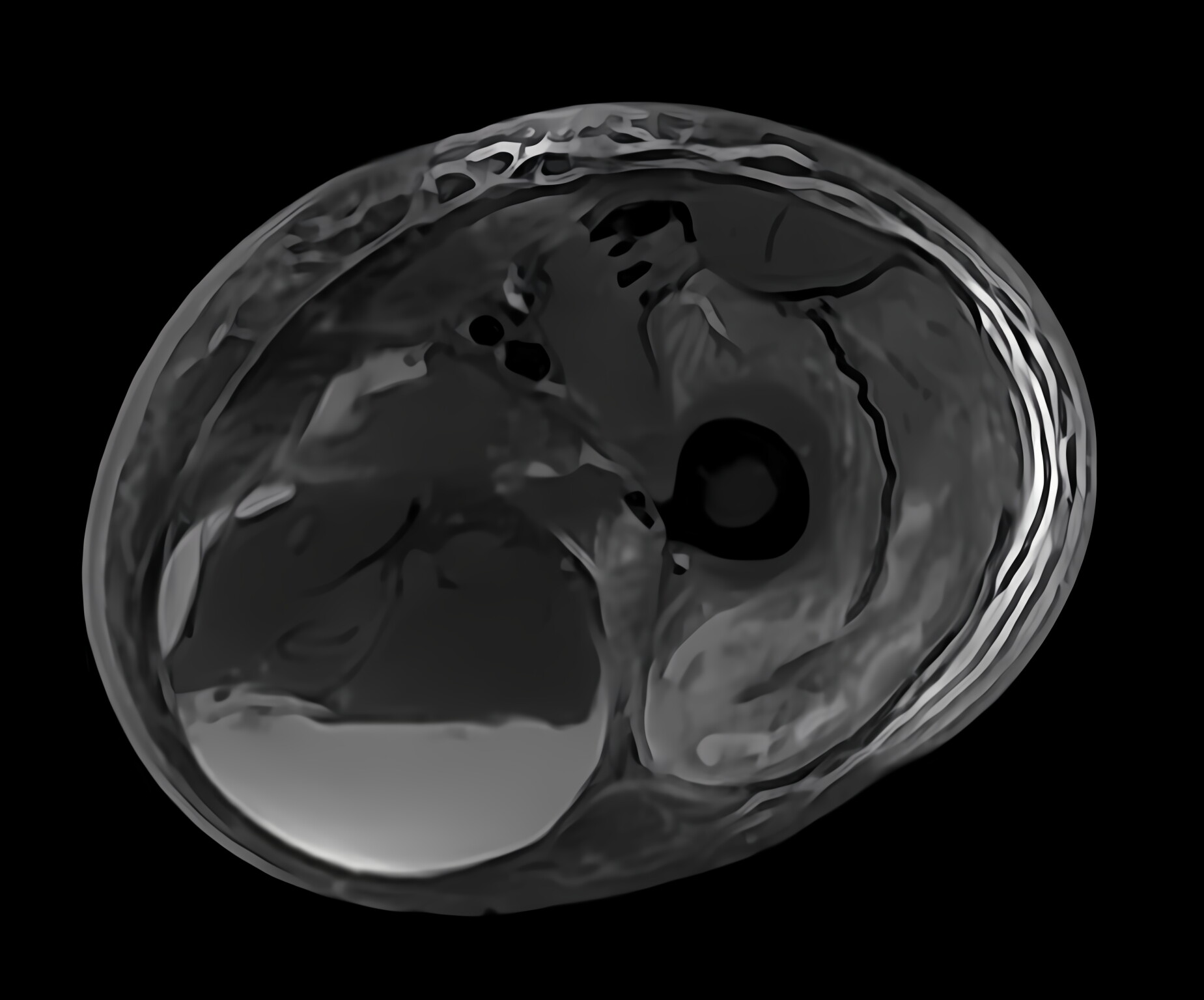

Supplement: Supplementary file 1 [file Image1.jpg]

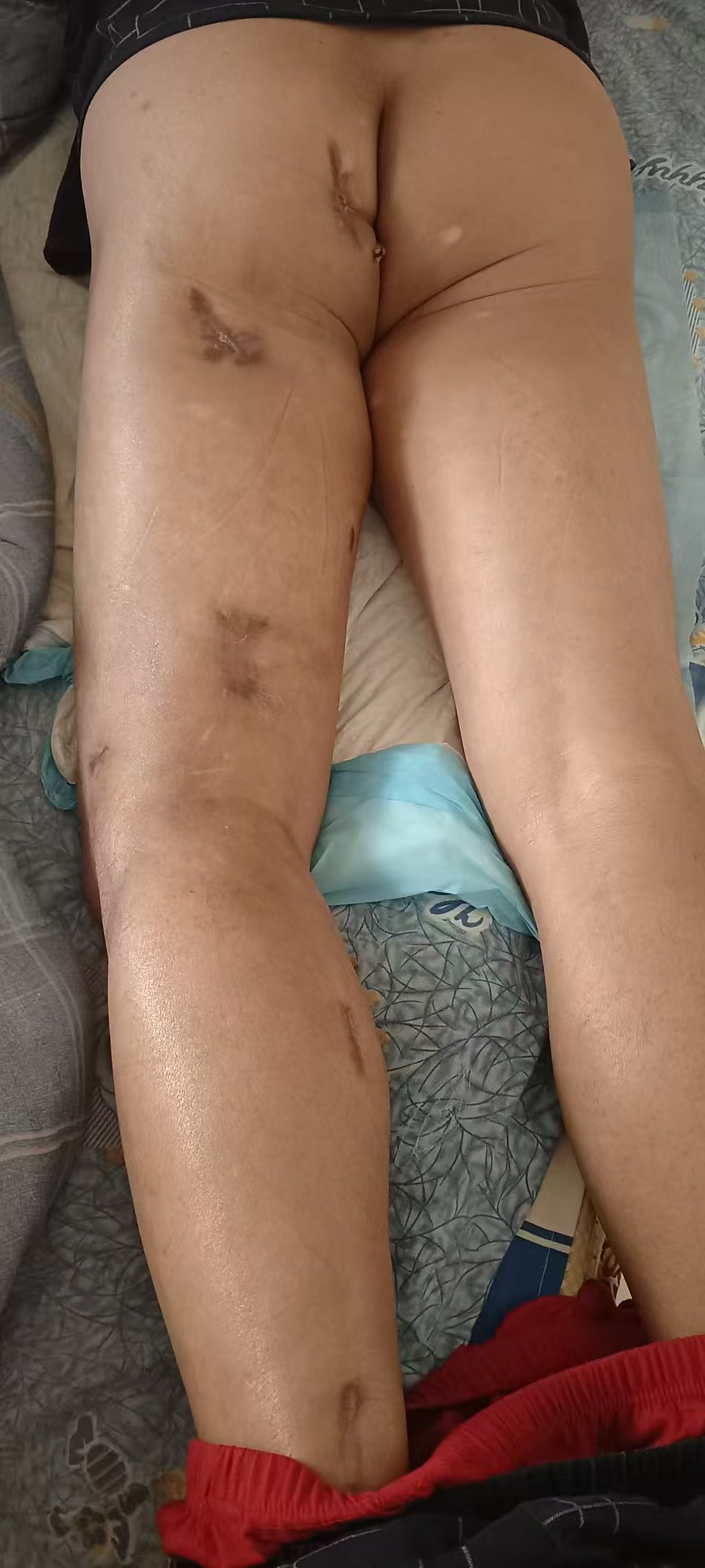

Supplement: Supplementary file 2 [file Image2.jpg]
